# Greener Surgery, Greener Hospital: Organizational Strategies for Carbon Footprint Reduction in Anesthesia and Perioperative Departments

**DOI:** 10.7759/cureus.116338

**Published:** 2026-09-16

**Authors:** Maria Bourazani, Maria S Chrysi, Despoina Pappa, Sofia Poulopoulou, Dimitris P Korkolis

**Affiliations:** 1 Department of Anesthesiology, General Anticancer and Oncology Hospital of Athens "Saint Savvas", Athens, GRC; 2 Department of Surgery, General Anticancer and Oncology Hospital of Athens "Saint Savvas", Athens, GRC; 3 Nursing, Erikos Ntinan Hospital, Athens, GRC; 4 Department of Surgical Oncology, General Anticancer and Oncology Hospital of Athens "Saint Savvas", Athens, GRC

**Keywords:** anesthesia, carbon footprint, greener surgery, organizational strategies, perioperative

## Abstract

Environmental sustainability has become an increasingly important priority in healthcare, particularly in anesthesia and perioperative services, where energy use, anesthetic gases, disposable materials, procurement, and waste contribute substantially to environmental burden. This narrative review examines how isolated environmental practices can be transformed into structured organizational strategies that can support sustained implementation and wider organizational adoption.

A structured literature search was conducted in PubMed/MEDLINE and Scopus between May and August 2026, followed by relevance assessment and narrative synthesis. Three complementary search domains addressed perioperative environmental sustainability, organizational implementation and governance, and hospital-level integration and scalability. Across both databases, 1,668 records were identified before deduplication, yielding 1,045 unique records for relevance assessment. Following relevance assessment and secondary appraisal, 40 publications were included in the final narrative synthesis. Evidence was synthesized across environmental impact, sustainable practices, organizational implementation, governance and measurement, and institutional scalability.

The literature indicates that interventions such as reduced anesthetic-gas use, low-flow anesthesia, waste segregation, reusable equipment, supply optimization, and energy conservation can reduce environmental impact. However, durable improvement depends on organizational mechanisms including leadership, defined accountability, multidisciplinary engagement, standardized workflows, procurement alignment, education, audit, and performance measurement. Departmental initiatives in anesthesia and perioperative care may therefore serve as practical implementation environments in which effective strategies can be evaluated, standardized, and adapted across hospital services.

Environmental sustainability in perioperative care should progress beyond isolated "green" practices toward integration within clinical governance and quality-improvement systems. The long-term objective is not only a greener operating room, but an organizational capacity for continuous environmental improvement while maintaining patient safety, quality of care, operational effectiveness, and responsible resource use.

## Introduction and background

Healthcare delivery contributes substantially to global greenhouse gas emissions through energy consumption, procurement, transportation, pharmaceuticals, infrastructure, and waste generation [1,2]. Within hospitals, operating theaters and perioperative services are particularly resource-intensive because of continuous ventilation, energy-demanding equipment, extensive use of disposable materials, pharmaceutical consumption, and anesthetic gases [3,4].

Anesthesia has therefore become an important focus of healthcare decarbonization. Recommended measures include reducing or eliminating the use of high-impact volatile anesthetics, minimizing fresh gas flow, avoiding unnecessary nitrous oxide, selecting reusable equipment when supported by lifecycle evidence, optimizing material use, and improving energy efficiency [5,6]. Broader operating-room sustainability initiatives additionally address recycling, supply optimization, procurement practices, and avoidance of unnecessary opening or disposal of consumables [7,8].

Despite the availability of established environmental interventions, their sustained integration into routine care remains inconsistent. Evidence indicates that successful implementation depends on organizational factors such as leadership, defined accountability, multidisciplinary engagement, standardized workflows, education, procurement alignment, measurement, and feedback [9-11]. Environmental sustainability should therefore be approached not solely as an individual professional responsibility, but as an organizational function integrated with quality improvement and clinical governance.

Anesthesia and perioperative departments provide a particularly relevant setting for this transition. Their activities are measurable, resource-intensive, highly standardized, and interconnected with surgery, nursing, pharmacy, sterile processing, procurement, facilities management, and hospital administration. Departmental sustainability programs may consequently function as implementation laboratories from which effective practices can be evaluated, standardized, and adapted across other hospital services.

Existing reviews have primarily characterized individual environmental interventions, implementation approaches, or barriers to sustainable operating-room practice [7,9,10]. The present review extends this literature by integrating these clinical and implementation perspectives with governance, leadership, procurement, measurement, workforce engagement, and hospital-level scalability, with particular emphasis on how isolated environmental actions can be transformed into durable organizational strategy.

This narrative review examines how environmental sustainability practices in anesthesia and perioperative care can evolve into structured organizational strategies. Particular attention is given to implementation, governance, leadership, measurement, multidisciplinary engagement, and the potential for departmental initiatives to contribute to broader hospital-level sustainability.

## Review

Methodology

Study Design

A narrative review design was adopted to integrate heterogeneous evidence spanning environmental assessment, clinical practice, implementation science, quality improvement, and organizational governance. This approach was selected because the review aimed to examine not only the environmental effects of sustainability interventions but also the organizational mechanisms that influence their implementation, durability, and scalability. Evidence selection was guided by relevance to the review question, recency, and applicability to perioperative sustainability and organizational implementation rather than by quantitative effect estimation.

Literature Search Strategy

A structured literature search was conducted in PubMed/MEDLINE and Scopus between May and August 2026. Three complementary search domains were developed to capture: (1) environmental sustainability in anesthesia and perioperative care; (2) organizational implementation, leadership, governance, and quality improvement; and (3) hospital-level integration and scalability.

PubMed searches were conducted using Title/Abstract fields, whereas Scopus searches used Title, Abstract, and Keywords. Search concepts included environmental sustainability, carbon footprint, green healthcare, anesthesia/anaesthesia, perioperative care, operating room/operating theatre, implementation, organizational/organisational change, strategy, governance, leadership, and quality improvement. The complete Boolean search strings, including database-specific field tags and operators, together with the number of records retrieved from each search, are presented in Table 1.

**Table 1:** Literature Search Strategy The six searches yielded 1,668 records before deduplication. Duplicates were identified using digital object identifiers (DOIs) and PubMed identifiers (PMIDs), supplemented by normalized exact-title matching when unique identifiers were unavailable. After deduplication, 1,045 unique records remained for relevance assessment.

Database	Search domain	Exact search strategy	Records retrieved
PubMed/MEDLINE	Environmental sustainability in anesthesia and perioperative care	("environmental sustainability"[Title/Abstract] OR "carbon footprint"[Title/Abstract] OR "green healthcare"[Title/Abstract]) AND (anesthesia[Title/Abstract] OR anaesthesia[Title/Abstract] OR perioperative[Title/Abstract] OR "operating room"[Title/Abstract] OR "operating theatre"[Title/Abstract])	198
PubMed/MEDLINE	Organizational implementation	("environmental sustainability"[Title/Abstract] OR "carbon footprint"[Title/Abstract] OR "green healthcare"[Title/Abstract]) AND (anesthesia[Title/Abstract] OR anaesthesia[Title/Abstract] OR perioperative[Title/Abstract] OR "operating room"[Title/Abstract] OR "operating theatre"[Title/Abstract]) AND (implementation[Title/Abstract] OR organizational[Title/Abstract] OR organisational[Title/Abstract] OR strategy[Title/Abstract] OR "quality improvement"[Title/Abstract] OR governance[Title/Abstract] OR leadership[Title/Abstract])	145
PubMed/MEDLINE	Hospital-level integration and scalability	("environmental sustainability"[Title/Abstract] OR "green healthcare"[Title/Abstract] OR "carbon footprint"[Title/Abstract]) AND (hospital[Title/Abstract] OR healthcare[Title/Abstract]) AND ("organizational change"[Title/Abstract] OR "organisational change"[Title/Abstract] OR governance[Title/Abstract] OR implementation[Title/Abstract] OR leadership[Title/Abstract] OR "quality improvement"[Title/Abstract])	218
Scopus	Environmental sustainability in anesthesia and perioperative care	TITLE-ABS-KEY(("environmental sustainability" OR "carbon footprint" OR "green healthcare") AND (anesthesia OR anaesthesia OR perioperative OR "operating room" OR "operating theatre"))	599
Scopus	Organizational implementation	TITLE-ABS-KEY(("environmental sustainability" OR "carbon footprint" OR "green healthcare") AND (anesthesia OR anaesthesia OR perioperative OR "operating room" OR "operating theatre") AND (implementation OR organizational OR organisational OR strategy OR "quality improvement" OR governance OR leadership))	152
Scopus	Hospital-level integration and scalability	TITLE-ABS-KEY(("environmental sustainability" OR "green healthcare" OR "carbon footprint") AND (hospital OR healthcare) AND ("organizational change" OR "organisational change" OR governance OR implementation OR leadership OR "quality improvement"))	356

The three PubMed searches retrieved 198, 145, and 218 records, respectively, while the corresponding Scopus searches retrieved 599, 152, and 356 records, yielding a total of 1,668 records across both databases.

Duplicates were removed using a hierarchical approach based primarily on digital object identifiers (DOIs) and PubMed identifiers (PMIDs), supplemented by normalized exact-title matching when unique identifiers were unavailable. After deduplication, 1,045 unique records remained for relevance assessment.

The review prioritized contemporary evidence, particularly publications from 2021 to 2026, while earlier seminal publications were retained when required to support established concepts in environmental impact assessment, lifecycle analysis, clinical governance, quality measurement, and organizational change.

Study Selection

Titles and abstracts of the 1,045 unique records were assessed for relevance to the review question. Priority was given to systematic and scoping reviews, consensus statements and professional guidance, implementation studies, quality-improvement initiatives, lifecycle assessments, observational studies, and organizational research directly addressing anesthesia, perioperative care, operating-room sustainability, or hospital environmental performance.

Evidence from other resource-intensive clinical settings was retained when it provided transferable insight into implementation mechanisms, governance structures, leadership, procurement, measurement, or institutional scalability. Publications were excluded when environmental sustainability was peripheral to the study objective or when the findings did not meaningfully inform perioperative or organizational sustainability.

Records retained after title and abstract screening underwent a second-stage appraisal using predefined relevance criteria. Publications were included when they met at least one of the following criteria: (1) directly addressed environmental sustainability in anesthesia, perioperative care, or operating-room practice; (2) evaluated implementation mechanisms, governance, leadership, procurement, measurement, or quality-improvement processes relevant to sustainability; (3) provided lifecycle, environmental, or organizational evidence applicable to perioperative services; or (4) offered transferable evidence from other resource-intensive hospital settings relevant to institutional scalability. Publications were excluded when sustainability was peripheral to the main study objective, when findings had limited applicability to perioperative or organizational sustainability, or when they substantially duplicated evidence already represented by more directly relevant or methodologically informative sources. Foundational publications outside the main search period were retained only when required to support established concepts in clinical governance, quality measurement, lifecycle assessment, or organizational change.

Following deduplication, 1,045 unique records underwent title and abstract relevance screening. Of these, 801 records were excluded because they did not meet the predefined relevance criteria, while 244 records were retained for further appraisal. The retained records comprised 57 records classified as core evidence, 107 as supportive evidence, and 80 as potentially relevant. These records subsequently underwent second-stage appraisal according to the inclusion and exclusion criteria described above, with consideration of direct thematic relevance, applicability to organizational implementation, and avoidance of redundant evidence. As this was a narrative review rather than a systematic review, formal independent duplicate screening and adjudication procedures were not undertaken. Following this process, 40 publications were included in the final narrative synthesis.

The resulting evidence base and its contribution to the principal thematic domains of the review are summarized in Table 2.

As this was a narrative rather than a systematic review, no formal risk-of-bias assessment, meta-analysis, or quantitative evidence synthesis was undertaken.

**Table 2:** Evidence Base and Thematic Contribution to the Narrative Synthesis The table summarizes the principal categories of evidence used to structure the narrative synthesis. It is intended to illustrate the methodological contribution of the evidence base rather than provide an exhaustive listing of included publications.

Thematic domain	Principal evidence considered	Contribution to the synthesis
Environmental footprint of anesthesia and perioperative care	Systematic reviews, lifecycle assessments, health-system carbon assessments, and consensus guidance	Defined the principal environmental hotspots of perioperative care, including anesthetic gases, energy use, consumables, procurement, waste, and equipment lifecycle impacts.
Sustainable clinical and operational practices	Quality-improvement studies, departmental interventions, systematic and scoping reviews	Identified practical interventions involving anesthetic-gas reduction, low-flow anesthesia, energy conservation, waste segregation, recycling, source reduction, reusable equipment, and supply optimization.
Organizational implementation	Implementation studies, structured quality-improvement programs, implementation frameworks, and departmental case studies	Demonstrated how environmental actions can be translated into standardized workflows, assigned responsibilities, staff education, monitoring, and structured departmental programs.
Governance, leadership, and measurement	Clinical governance frameworks, organizational studies, leadership research, and sustainability-metrics reviews	Supported integration of sustainability into governance structures, leadership accountability, multidisciplinary collaboration, performance measurement, audit, feedback, and continuous improvement.
Departmental-to-hospital scalability	Hospital-level organizational studies, cross-specialty sustainability models, and large-scale implementation studies	Informed the transition from departmental initiatives to broader institutional strategies through governance integration, shared metrics, procurement alignment, and interdepartmental adaptation.
Implementation barriers and safety considerations	Systematic reviews, qualitative studies, professional surveys, pathway analyses, and evidence-informed policy guidance	Identified barriers related to workload, infrastructure, procurement, organizational support, clinical culture, infection concerns, and the need to assess environmental benefit alongside patient safety and clinical outcomes.

Thematic synthesis

Selected evidence was synthesized narratively across five interconnected domains: environmental footprint, sustainable clinical practices, organizational implementation, governance and measurement, and hospital-level scalability.

For implementation-focused studies, three dimensions were considered consistently: the environmental effect of the intervention, the organizational mechanisms supporting its implementation, and its potential for transfer or scale-up. Particular attention was given to leadership, defined accountability, multidisciplinary participation, standardized workflows, procurement alignment, education, audit, feedback, and performance indicators.

The synthesis followed a progressive organizational pathway in which individual environmental practices are first incorporated into standardized departmental processes, subsequently integrated into governance and continuous measurement systems, and ultimately adapted across departments to support broader hospital sustainability.

This framework was used to distinguish isolated environmental actions from interventions with the potential to become durable components of routine organizational practice.

The Environmental Footprint of Anesthesia and Perioperative Care

The environmental burden of perioperative care extends across the entire clinical pathway and includes direct emissions, energy consumption, pharmaceuticals, disposable supplies, equipment manufacture and reprocessing, waste treatment, and procurement-related emissions [4,6,12]. Consequently, environmental assessment should consider both direct clinical activities and the upstream and downstream effects of resource use [3,4,6,7].

Anesthetic gases constitute a distinctive source of direct greenhouse gas emissions because their atmospheric effects vary substantially among agents [13]. Current recommendations favor avoidance of high-impact agents when clinically appropriate, reduction of fresh gas flows, limitation of nitrous oxide use, and consideration of intravenous or regional techniques where suitable [5,6]. For nitrous oxide, however, environmental improvement can result from two distinct mechanisms: reducing clinical administration and reducing losses within the gas supply system. The former reflects changes in anesthetic practice and reduced patient-level use, whereas the latter involves minimizing avoidable losses associated with central supply infrastructure, manifolds, pipelines, cylinders, and leakage where these systems remain in use [5,6]. Department- and hospital-level initiatives should therefore distinguish reductions in clinical consumption from reductions attributable to supply-system optimization, as both can contribute to lower overall nitrous oxide-related emissions [5,6]. More broadly, initiatives targeting volatile anesthetics and nitrous oxide have demonstrated measurable reductions in carbon dioxide-equivalent emissions when implemented systematically [14-16].

Energy use represents another major contributor. Operating rooms require continuous ventilation, temperature control, lighting, and equipment availability, including during periods of limited clinical activity. Structured power-down interventions have shown that energy consumption can be reduced through defined shutdown procedures and clear assignment of responsibility without altering clinical care [17].

The environmental impact of materials extends beyond visible operating-room waste. Single-use products generate emissions through raw-material extraction, manufacture, packaging, transportation, and disposal [3,7]. Procurement decisions should therefore incorporate lifecycle considerations and opportunities for source reduction rather than focus solely on acquisition cost or end-of-life waste.

Reusable equipment may reduce material consumption and waste, but its environmental advantage depends on product lifespan, sterilization requirements, water and energy use, transportation, and the local energy mix [18]. Recent large-scale analyses nevertheless suggest substantial opportunities for reducing both waste and expenditure when reusable alternatives are clinically appropriate and supported by effective reprocessing systems [19].

Taken together, these findings support a systems-based approach to perioperative sustainability. Anesthetic gases, energy, materials, procurement, and reprocessing should be assessed as interconnected components of the same environmental footprint rather than as independent targets for isolated intervention [3,4,6,7,18,19].

From Green Practices to Organizational Strategy

The principal challenge in perioperative sustainability is no longer the identification of individual interventions but their conversion into durable organizational practice. A systematic review of implementation approaches in operating rooms found that most sustainability initiatives remained at exploratory or early implementation stages and that established implementation frameworks were rarely used [9]. This implementation gap helps explain why effective environmental interventions may remain dependent on individual motivation rather than becoming routine practice [9-11].

Organizational integration requires defined responsibilities, standardized procedures, multidisciplinary engagement, education, procurement alignment, and measurable objectives. Systematic and scoping reviews demonstrate that waste reduction, recycling, supply optimization, anesthetic-gas reduction, energy conservation, and reuse can generate environmental and economic benefits when incorporated into structured improvement programs [7,8,10].

Waste management provides a clear example. Staff education followed by structured waste segregation has produced measurable reductions in operating-room waste [20]. Similarly, rationalizing disposable supplies and selecting reusable alternatives can shift sustainability upstream from waste management toward source reduction and procurement [19]. These approaches are organizational rather than purely behavioral because they require changes in supply systems, purchasing decisions, storage, workflow, and accountability [9,10,19].

Anesthetic practice offers comparable opportunities. Electronic clinical decision support has been used to reinforce low fresh gas flow within routine workflow, demonstrating how an environmental objective can be translated from professional recommendation into a system-level intervention [21]. Departmental strategies targeting nitrous oxide and volatile anesthetics likewise demonstrate that environmental improvement can be implemented and measured across entire clinical services rather than relying on isolated clinician choices [14-16].

A practical example is provided by the implementation of a 5R program comprising Recycle, Rethink, Research, Reduce, and Reuse within the Anesthesiology and Surgery Departments of a Greek oncology hospital [22]. The program combined waste segregation, rationalization of surgical sets, reduction of pharmaceutical and material waste, low-flow anesthesia, avoidance of nitrous oxide, reusable technologies where appropriate, energy-saving measures, and staff education. A total of 12 nurses from two departments were designated to reinforce the new procedures until they became incorporated into everyday practice, providing an explicit implementation mechanism beyond individual professional commitment [22].

Importantly, implementation was accompanied by measurable outcomes. Despite a 6.17% increase in the number of operations, total annual desflurane consumption decreased from 915.60 bottles in 2022 to 901 bottles in 2023, corresponding to a 1.59% reduction. Over the same period, desflurane consumption per procedure decreased from 69.848 mL to 64.742 mL, representing a 7.31% reduction [22]. Although observational and single-center, this experience illustrates the central concept of the present review: environmental practices become organizational strategy when they are coordinated, assigned, monitored, and integrated into routine operations [22].

Structured tools can support this transition. Healthcare Sustainability Mode and Effect Analysis (HSMEA), adapted from Healthcare Failure Mode and Effect Analysis principles, provides a systematic method for identifying environmentally significant processes, prioritizing interventions, and embedding sustainability within improvement systems [23]. The objective is therefore not to accumulate independent "green" actions, but to develop a coherent departmental program with defined governance, standardized processes, and measurable outcomes [9,23].

Procurement, Circularity, and Resource Stewardship

Environmental sustainability in perioperative care extends beyond waste segregation and end-of-life disposal. A substantial proportion of healthcare-related environmental impact is embedded upstream in the manufacture, packaging, transportation, and procurement of pharmaceuticals, devices, textiles, and other clinical supplies [3,7,18]. Consequently, organizational sustainability strategies should increasingly prioritize source reduction and lifecycle-informed procurement rather than relying predominantly on recycling [7,18,19].

Procurement policies can influence environmental performance by incorporating product durability, reusability, packaging, transportation requirements, reprocessing infrastructure, and total lifecycle cost into purchasing decisions [7,18,19]. This approach is particularly relevant in operating rooms, where standardized procedure packs and routine opening of potentially unnecessary consumables may generate avoidable material use. Reviewing pack composition, matching supplies to actual procedural requirements, and preventing unnecessary opening of sterile products can reduce both resource consumption and expenditure [7,19,22].

The transition from single-use to reusable devices nevertheless requires contextual assessment. Reusable products are not intrinsically environmentally superior; their overall impact depends on lifespan, sterilization processes, water and energy consumption, transportation, and the carbon intensity of local energy systems [18]. Organizational procurement decisions should therefore integrate environmental lifecycle evidence with infection prevention, clinical performance, operational feasibility, and cost rather than applying generalized assumptions regarding disposability or reuse [18,24].

Circularity further extends beyond product selection. Redistribution of unused materials, inventory optimization, equipment maintenance, and prevention of premature disposal can retain the value of resources within healthcare systems [22]. Embedding these practices within procurement and financial governance can shift sustainability from waste management toward broader resource stewardship [7,19,22].

Governance, Leadership, and Measurement for Sustainable Change

Environmental sustainability becomes durable when incorporated into governance and quality-management systems. Clinical governance provides an appropriate framework because it links organizational responsibility, professional accountability, quality improvement, and continuous evaluation [25]. Sustainability can therefore be managed as a dimension of healthcare performance rather than as an optional environmental activity [25].

The structure-process-outcome framework offers a practical basis for measurement [26]. Structural measures may include designated sustainability leadership, multidisciplinary teams, procurement standards, education programs, and institutional policies. Process indicators can assess adherence to low-flow anesthesia, waste segregation, reusable-device pathways, or equipment power-down procedures. Outcomes may include greenhouse-gas emissions, material consumption, waste, energy use, cost, and relevant clinical outcomes.

Measurement remains a methodological weakness in the field. Sustainability quality-improvement publications use heterogeneous metrics, with global warming potential predominating and less consistent assessment of resource use, waste, water, toxicity, or broader lifecycle effects [27]. Standardized indicators are therefore necessary if interventions are to be compared, monitored longitudinally, or scaled across institutions.

Leadership influences whether sustainability becomes operational. Implementation studies identify managerial priority, dedicated implementation leads, multidisciplinary working groups, education, and feedback as important facilitators [11]. Formal accountability also appears relevant: departments in which sustainability is incorporated into leadership responsibilities report greater sustainability activity [28].

Nursing leadership has particular relevance in perioperative settings because nurses influence material use, medication preparation, waste segregation, equipment management, workflow standardization, and team engagement [29]. The designated nursing roles reported by Chrysi et al. provide a practical example of distributing implementation responsibility within the clinical workforce rather than restricting sustainability to senior management [22].

Governance should therefore connect environmental performance with existing systems for quality, safety, procurement, finance, and operational management. Sustainability indicators should be reviewed alongside clinical and economic outcomes, creating a continuous cycle of measurement, feedback, corrective action, and reassessment [25-28].

Sustainability Metrics and Data Infrastructure

The transition from environmental initiatives to organizational strategy requires reliable measurement. Without standardized indicators, sustainability activities risk remaining episodic and difficult to evaluate consistently over time. Existing healthcare sustainability studies employ heterogeneous metrics, with carbon dioxide-equivalent emissions and global warming potential receiving considerably greater attention than other environmental dimensions such as waste, water use, toxicity, and material depletion [27].

A practical measurement framework should incorporate structural, process, and outcome indicators [26]. Structural indicators may include designated sustainability leadership, formal policies, multidisciplinary governance structures, education programs, and procurement standards. Process indicators can measure adherence to low-flow anesthesia, waste segregation, reusable-device pathways, power-down procedures, or supply optimization. Outcome measures may include anesthetic consumption, CO2-equivalent emissions, waste weight, material utilization, electricity use, expenditure, and relevant clinical outcomes [17,21,22,27].

For routine organizational monitoring, CO₂-equivalent emissions may be most meaningfully assessed longitudinally within the same department or institution, using a consistent accounting boundary and methodology over time. The emission sources included should be explicitly defined and measured using the same published conversion factors or global warming potential values in each reporting period. Annual absolute emissions should be interpreted alongside an activity-based denominator, such as CO₂e per procedure, operating-room hour, or patient episode, to account for changes in clinical volume. This approach allows each department to assess year-on-year improvement against its own baseline while avoiding potentially misleading comparisons between departments or institutions with different case mix, activity levels, infrastructure, and service profiles.

Digital infrastructure may strengthen this process. Electronic clinical decision support has already demonstrated the potential to embed environmental objectives directly into routine anesthetic workflow by prompting lower fresh gas flows and enabling measurable changes in practice [21]. Similar approaches could support automated dashboards, longitudinal performance tracking, audit cycles, and feedback to clinical teams.

However, environmental indicators should not be interpreted independently. A reduction in carbon emissions or material use cannot be considered successful if it compromises patient safety, increases downstream healthcare utilization, or transfers environmental burden elsewhere in the care pathway [18,30]. Sustainability measurement should therefore be integrated with clinical quality, safety, operational performance, and economic outcomes [27-30].

Workforce Engagement, Education, and Organizational Culture

Sustainability strategies depend on the behavior of clinicians, nurses, managers, procurement professionals, and technical staff who collectively shape resource use. Surveys and implementation studies demonstrate that favorable attitudes toward environmental sustainability do not consistently translate into routine practice, particularly when staff face workload pressures, limited training, inadequate infrastructure, or weak organizational support [10,31,32].

Education is therefore necessary but insufficient when delivered as an isolated intervention. Training is more likely to influence practice when combined with clearly defined responsibilities, accessible procedures, feedback mechanisms, local champions, and visible managerial support [9-11]. Sustainability should therefore be embedded within routine professional practice rather than remain an additional voluntary responsibility dependent on highly motivated individuals [9-11].

Perioperative nurses have a particularly important role because they influence medication preparation, equipment use, waste segregation, supply management, workflow organization, and communication across professional groups [29]. Designating nurses or other clinicians as sustainability leads can distribute implementation responsibility and reinforce new practices during the transition from project-based interventions to routine care [22].

Organizational culture also influences the acceptability of environmental change. Established preferences, perceived infection risks, procurement conventions, and professional norms can impede adoption even when environmentally preferable alternatives are available [24,33]. Successful implementation therefore requires attention to both formal governance and informal clinical culture. Leadership endorsement, multidisciplinary co-design, transparent safety evaluation, and feedback on measurable outcomes may help sustainability become embedded as a legitimate component of healthcare quality rather than perceived as an external environmental agenda [10,11,24,34].

From the Anesthesia Department to the Greener Hospital

Departmental sustainability programs can provide a practical route toward broader institutional transformation. Anesthesia is particularly suitable for pilot implementation because its workflows are standardized, resource consumption is measurable, and its operations intersect with multiple clinical and non-clinical services [5,6,22].

However, scalability should not be understood as simple replication. Successful diffusion requires interventions to be evaluated locally, standardized where appropriate, supported by common metrics, and integrated with procurement, infrastructure, workforce, and governance systems. Hospital-level evidence suggests that environmental activity frequently develops before formal strategic commitment, emphasizing the importance of subsequently embedding successful initiatives within organizational policy and accountability structures [35].

The 5R program reported by Chrysi et al. illustrates the beginning of this transition. Although initiated within anesthesia and surgery, implementation required support from hospital administration and the financial department, while materials were redistributed through pharmacy and procurement channels to other clinical departments and hospitals [22]. Its significance therefore extends beyond the individual environmental interventions and lies in the development of cross-departmental operational relationships [22].

Comparable models have emerged in other resource-intensive specialties. Interventional radiology has proposed incorporation of environmental indicators into departmental quality-improvement programs [36], while interventional cardiology demonstrates how procurement, waste, energy, and multidisciplinary practice can be addressed through a structured sustainability framework [37]. Hospital-wide recycling initiatives similarly demonstrate that locally developed programs can expand beyond their original department when organizational infrastructure supports diffusion [38].

Large-scale implementation studies in anesthesia further indicate that environmental strategies can be institutionalized across multiple facilities rather than remaining confined to a single department [39]. More broadly, hospital sustainability research emphasizes governance, organizational commitment, infrastructure, and strategic alignment as prerequisites for systemic implementation [34].

The pathway from greener anesthesia to a greener hospital can therefore be conceptualized as an organizational progression: pilot intervention, measurement, standardization, governance integration, interdepartmental adaptation, and institutional strategy [22,34-39].

Anesthesia need not be considered the sole or universally optimal starting point; rather, it can serve as a high-impact catalyst from which transferable organizational capabilities are developed [34,35,39].

Implementation Challenges and Safety Considerations

Implementation remains constrained by competing clinical priorities, workload, insufficient training, inadequate infrastructure, procurement restrictions, financial concerns, and limited organizational support [10,31,33]. Surveys consistently demonstrate a gap between favorable attitudes toward sustainability and routine environmental behavior, indicating that professional motivation alone is insufficient [31,32].

Procurement and established clinical culture can reinforce unsustainable practices. Qualitative evidence concerning reusable surgical textiles identifies convenience, perceived costs, procurement complexity, infection concerns, and limited authority among staff in sustainability roles as important barriers to change [24]. Implementation therefore requires both evidence and organizational capacity [10,24,31-34].

Patient safety remains non-negotiable. Decisions concerning reuse, reprocessing, ventilation, pharmaceutical practice, or altered perioperative pathways should be evaluated within established infection-prevention, risk-management, and quality-assurance systems. Reusable technologies may offer environmental advantages, but equipment durability, reprocessing requirements, performance, and local infrastructure must be considered [18,24].

Environmental benefit should also be assessed across the entire care pathway. Lower-resource pathways may reduce immediate emissions while generating additional downstream care if associated with complications or readmission. Comparative pathway analyses illustrate why carbon reduction cannot be evaluated independently of clinical outcomes [30].

Sustainable implementation must therefore satisfy a multidimensional test: environmental benefit, patient safety, clinical effectiveness, operational feasibility, and economic responsibility [18,24,30,40].

Recent evidence-informed policy guidance for operating-room sustainability similarly emphasizes that environmental interventions should be integrated with established standards of patient safety, clinical effectiveness, and quality improvement rather than implemented as parallel or isolated objectives [40]. This approach avoids the false assumption that environmental performance and healthcare quality are competing objectives [40].

The principal environmental sustainability interventions identified across anesthesia and perioperative care are summarized in Table 3. The table links each intervention with its primary environmental target, key organizational requirements, and measurable outcomes, providing an integrated overview of the clinical, operational, and governance measures discussed throughout this review.

**Table 3:** Key Environmental Sustainability Interventions and Organizational Strategies in Anesthesia and Perioperative Care Intervention selection should be guided by clinical appropriateness, patient safety, local infrastructure, lifecycle evidence, and organizational feasibility. Environmental performance should be assessed together with clinical, operational, and economic outcomes. Sustainability interventions should therefore be incorporated into established quality-improvement and governance systems rather than implemented as isolated environmental activities.

Intervention / strategy	Primary environmental target	Key organizational requirements	Potential measurable outcomes	Representative references
Reduction or avoidance of high-impact volatile anesthetics	Direct greenhouse-gas emissions	Departmental policy, formulary alignment, clinician education, monitoring of anesthetic-agent use	Volatile anesthetic consumption; CO₂-equivalent emissions	[5,6,14-16]
Low-flow anesthesia	Anesthetic-gas consumption and emissions	Standardized protocols, staff training, decision support, monitoring	Fresh gas flow rates; anesthetic consumption; CO₂-equivalent emissions	[5,6,21]
Reduction of nitrous oxide clinical use and supply-system waste	Direct greenhouse-gas emissions	Clinical guidance, review of actual clinical demand, assessment of central supply infrastructure, leak identification, and consideration of cylinder-based alternatives or supply-system rationalization where appropriate	Clinical nitrous oxide use; total nitrous oxide purchased or supplied; discrepancy between supply and documented clinical use; CO₂-equivalent emissions	[5,6,15,16]
Use of intravenous or regional techniques when clinically appropriate	Anesthetic-related emissions	Clinical protocols, professional competence, equipment and drug availability, patient-specific assessment	Anesthetic-related emissions; relevant clinical outcomes	[5,6]
Operating-room energy optimization and power-down procedures	Electricity consumption	Defined shutdown protocols, assigned responsibilities, coordination with facilities management	Electricity consumption; energy cost; estimated emissions	[17]
Waste segregation and recycling	Waste generation and disposal	Clearly defined waste streams, staff education, signage, monitoring, waste-management coordination	Waste weight/volume; recycling rate; disposal costs	[7,8,20]
Source reduction and avoidance of unnecessary opening of consumables	Material consumption and supply-chain emissions	Procedure-specific standardization, pack review, multidisciplinary agreement, inventory management	Unused/opened items; waste volume; procurement expenditure	[7,8,19,22]
Rationalization of surgical and anesthesia packs	Material use and procurement	Review of pack composition, standardization, collaboration between clinical teams and procurement	Unused supplies; stock utilization; material expenditure	[7,22]
Replacement of selected single-use items with reusable alternatives	Material production, transport, and waste	Lifecycle assessment, reprocessing capacity, infection-prevention oversight, procurement support	Single-use items avoided; waste; cost; lifecycle emissions	[18,19,24]
Lifecycle-informed procurement	Upstream supply-chain emissions	Environmental procurement criteria, lifecycle evidence, collaboration among clinical, procurement, finance, and sustainability teams	Material consumption; lifecycle emissions; expenditure; product utilization	[7,18,19]
Inventory optimization and redistribution of unused materials	Avoidable procurement, material waste, and resource loss	Inventory surveillance, communication with pharmacy/procurement, redistribution pathways, stock accountability	Expired or unused stock; purchasing expenditure; redistributed materials	[22]
Electronic clinical decision support	Anesthetic-gas consumption and behavioral implementation	Integration into electronic workflow, clinician engagement, automated prompts, monitoring and feedback	Compliance with low-flow targets; fresh gas flow; anesthetic consumption	[21]
Staff education and competency development	Implementation reliability and environmental behavior	Structured education, repeated reinforcement, integration with clinical practice, feedback	Knowledge; compliance; intervention uptake; sustainability-related behavior	[10,11,20,22,29,31,32]
Designated sustainability leads or local champions	Organizational implementation and accountability	Defined roles, leadership support, protected responsibility, multidisciplinary communication	Intervention uptake; adherence; staff participation; implementation continuity	[9-11,22,28,29]
Multidisciplinary sustainability programs	Multiple environmental domains	Governance structure, shared objectives, clinical and non-clinical participation, measurement and feedback	Emissions; waste; material use; cost; implementation indicators	[9-11,22,23]
Integration of sustainability indicators into quality improvement	Continuous environmental performance improvement	Defined KPIs, audit cycles, feedback, governance oversight, linkage with clinical and economic outcomes	Environmental KPIs; adherence trends; clinical, operational, and economic outcomes	[23,25-28,40]
Sustainability dashboards and data-driven monitoring	Measurement, longitudinal performance tracking, and continuous improvement	Reliable data collection, defined indicators, digital infrastructure, reporting responsibility	Longitudinal emissions; resource use; waste; adherence; year-on-year departmental performance	[21,26,27]
Organizational governance and policy integration	Institutional sustainability and scalability	Executive support, accountability structures, integration with quality, safety, procurement, and finance	Policy adoption; departmental participation; institutional sustainability indicators	[25,28,34,35,40]
Interdepartmental adaptation and institutional scale-up	Hospital-wide environmental performance	Evaluation of pilot interventions, common metrics, governance integration, procurement alignment, adaptation to local workflows	Number of participating departments; institutional emissions/resource indicators; implementation sustainability	[22,34-39]
Safety-integrated environmental decision-making	Prevention of unintended clinical or environmental consequences	Infection-prevention oversight, clinical risk assessment, lifecycle evaluation, monitoring of downstream outcomes	Complications; readmissions; environmental outcomes; cost; operational performance	[18,24,30,40]

Limitations

This review has several limitations. First, its narrative design was selected to integrate heterogeneous evidence across environmental assessment, clinical practice, implementation science, quality improvement, and organizational governance, but it does not provide the methodological control of a systematic review. No formal risk-of-bias or study-quality assessment was undertaken, and the included evidence comprised a heterogeneous mixture of reviews, observational studies, quality-improvement initiatives, lifecycle assessments, surveys, implementation reports, and professional guidance. This heterogeneity limits direct comparison across studies and precludes quantitative synthesis or formal estimation of effect size. In addition, many sustainability studies are single-center, uncontrolled, or based on relatively short follow-up periods, and some rely on self-reported practices or attitudes. These factors reduce confidence in causal attribution, long-term sustainability, and generalizability across institutions with different infrastructure, case mix, procurement systems, energy sources, and organizational contexts. Accordingly, the conclusions of this review should be interpreted as evidence supporting the feasibility, organizational relevance, and potential value of structured sustainability strategies rather than as definitive evidence of their comparative or long-term effectiveness.

## Conclusions

The transition toward environmentally sustainable anesthesia and perioperative care requires more than the adoption of individual "green" practices. The evidence reviewed indicates that durable improvement depends on integrating environmental objectives into organizational structures, including governance, leadership, procurement, workforce engagement, standardized workflows, measurement, and quality-improvement systems.

Anesthesia and perioperative departments can provide a practical environment for testing and refining sustainability interventions, but broader impact depends on their adaptation across services and incorporation into institutional strategy. The ultimate objective is therefore not simply to reduce emissions, waste, or resource consumption within the operating room, but to develop an organizational capacity for continuous environmental improvement while maintaining patient safety, clinical effectiveness, operational feasibility, and responsible resource use.
